# Supplementary material for: Modeling glioblastoma heterogeneity as a dynamic network of cell states
Source: Mol Syst Biol. 2021 Sep 16;17(9):e10105. doi: 10.15252/msb.202010105 (PMC8444284; doi:10.15252/msb.202010105)
Supplement: Supplementary file 5 — Source Data for Figure 3 [file MSB-17-e10105-s001.zip › Figure3A_sourcedata/GSEA_3065/nosigdb_state5.GseaPreranked.1622718435914/RICHARDS_NOWAKOWSKI_SCIENCE_2017_OPC_UPREG.html]

Details for gene set RICHARDS\_NOWAKOWSKI\_SCIENCE\_2017\_OPC\_UPREG[GSEA]

|  || Dataset | state5 |
| Phenotype | NoPhenotypeAvailable |
| Upregulated in class | na\_pos |
| GeneSet | RICHARDS\_NOWAKOWSKI\_SCIENCE\_2017\_OPC\_UPREG |
| Enrichment Score (ES) | 0.52193934 |
| Normalized Enrichment Score (NES) | 2.4120433 |
| Nominal p-value | 0.0 |
| FDR q-value | 0.0 |
| FWER p-Value | 0.0 |
Table: GSEA Results Summary

  

Fig 1: Enrichment plot: RICHARDS\_NOWAKOWSKI\_SCIENCE\_2017\_OPC\_UPREG      
 Profile of the Running ES Score & Positions of GeneSet Members on the Rank Ordered List

  

| PROBE | GENE SYMBOL | GENE\_TITLE | RANK IN GENE LIST | RANK METRIC SCORE | RUNNING ES | CORE ENRICHMENT || 1 | KCNQ1OT1 |  |  | 8 | 0.931 | 0.0117 | Yes |
| 2 | NOVA1 |  |  | 10 | 0.870 | 0.0233 | Yes |
| 3 | PTPRZ1 |  |  | 12 | 0.862 | 0.0349 | Yes |
| 4 | CD9 |  |  | 14 | 0.857 | 0.0464 | Yes |
| 5 | BCAN |  |  | 15 | 0.850 | 0.0579 | Yes |
| 6 | PTN |  |  | 19 | 0.824 | 0.0687 | Yes |
| 7 | SOX6 |  |  | 28 | 0.754 | 0.0780 | Yes |
| 8 | PDIA6 |  |  | 29 | 0.742 | 0.0880 | Yes |
| 9 | GRIA2 |  |  | 32 | 0.732 | 0.0977 | Yes |
| 10 | HSP90B1 |  |  | 33 | 0.729 | 0.1075 | Yes |
| 11 | SCRG1 |  |  | 36 | 0.715 | 0.1170 | Yes |
| 12 | PLAT |  |  | 37 | 0.714 | 0.1266 | Yes |
| 13 | GPM6B |  |  | 41 | 0.702 | 0.1358 | Yes |
| 14 | HSPA5 |  |  | 43 | 0.698 | 0.1451 | Yes |
| 15 | PGRMC1 |  |  | 52 | 0.639 | 0.1529 | Yes |
| 16 | CANX |  |  | 53 | 0.634 | 0.1614 | Yes |
| 17 | GPM6A |  |  | 60 | 0.609 | 0.1690 | Yes |
| 18 | B2M |  |  | 61 | 0.601 | 0.1771 | Yes |
| 19 | PON2 |  |  | 84 | 0.546 | 0.1820 | Yes |
| 20 | FGFBP3 |  |  | 85 | 0.545 | 0.1893 | Yes |
| 21 | LAPTM4B |  |  | 97 | 0.532 | 0.1953 | Yes |
| 22 | LRRC4C |  |  | 101 | 0.525 | 0.2020 | Yes |
| 23 | CADM4 |  |  | 102 | 0.525 | 0.2091 | Yes |
| 24 | PLEKHH2 |  |  | 106 | 0.520 | 0.2158 | Yes |
| 25 | DSEL |  |  | 107 | 0.518 | 0.2228 | Yes |
| 26 | SEMA5A |  |  | 111 | 0.516 | 0.2295 | Yes |
| 27 | ITM2B |  |  | 117 | 0.509 | 0.2358 | Yes |
| 28 | ITGAV |  |  | 118 | 0.508 | 0.2427 | Yes |
| 29 | ITGB8 |  |  | 126 | 0.503 | 0.2487 | Yes |
| 30 | LSAMP |  |  | 132 | 0.497 | 0.2548 | Yes |
| 31 | SCARB2 |  |  | 134 | 0.494 | 0.2614 | Yes |
| 32 | PPIB |  |  | 156 | 0.477 | 0.2654 | Yes |
| 33 | DPP6 |  |  | 161 | 0.475 | 0.2714 | Yes |
| 34 | ABHD2 |  |  | 163 | 0.473 | 0.2777 | Yes |
| 35 | MMP16 |  |  | 165 | 0.470 | 0.2839 | Yes |
| 36 | SCD5 |  |  | 173 | 0.460 | 0.2893 | Yes |
| 37 | PDGFRA |  |  | 175 | 0.460 | 0.2954 | Yes |
| 38 | SPCS3 |  |  | 183 | 0.454 | 0.3008 | Yes |
| 39 | VCAN |  |  | 191 | 0.444 | 0.3060 | Yes |
| 40 | MAGI1 |  |  | 196 | 0.442 | 0.3115 | Yes |
| 41 | KIZ |  |  | 201 | 0.439 | 0.3170 | Yes |
| 42 | SLC44A1 |  |  | 216 | 0.428 | 0.3212 | Yes |
| 43 | MAML2 |  |  | 220 | 0.425 | 0.3266 | Yes |
| 44 | RDH11 |  |  | 224 | 0.422 | 0.3319 | Yes |
| 45 | ALCAM |  |  | 225 | 0.422 | 0.3376 | Yes |
| 46 | SERPINE2 |  |  | 238 | 0.414 | 0.3418 | Yes |
| 47 | SRPRB |  |  | 251 | 0.408 | 0.3460 | Yes |
| 48 | TMX1 |  |  | 255 | 0.406 | 0.3511 | Yes |
| 49 | LRP1 |  |  | 259 | 0.404 | 0.3563 | Yes |
| 50 | SCD |  |  | 284 | 0.391 | 0.3588 | Yes |
| 51 | PCDH9 |  |  | 285 | 0.390 | 0.3641 | Yes |
| 52 | HSD17B12 |  |  | 311 | 0.377 | 0.3663 | Yes |
| 53 | CHD7 |  |  | 312 | 0.376 | 0.3714 | Yes |
| 54 | DST |  |  | 313 | 0.376 | 0.3764 | Yes |
| 55 | TMX3 |  |  | 315 | 0.375 | 0.3814 | Yes |
| 56 | TRIO |  |  | 323 | 0.372 | 0.3856 | Yes |
| 57 | TMEM132B |  |  | 335 | 0.365 | 0.3893 | Yes |
| 58 | YIPF6 |  |  | 340 | 0.364 | 0.3938 | Yes |
| 59 | SETD5 |  |  | 360 | 0.358 | 0.3964 | Yes |
| 60 | DAG1 |  |  | 373 | 0.351 | 0.3998 | Yes |
| 61 | ERBB3 |  |  | 411 | 0.337 | 0.4001 | Yes |
| 62 | SEZ6L |  |  | 422 | 0.334 | 0.4035 | Yes |
| 63 | BCHE |  |  | 425 | 0.333 | 0.4077 | Yes |
| 64 | PCDHGC3 |  |  | 428 | 0.331 | 0.4120 | Yes |
| 65 | EMC10 |  |  | 429 | 0.331 | 0.4165 | Yes |
| 66 | UFL1 |  |  | 436 | 0.330 | 0.4202 | Yes |
| 67 | HAS2 |  |  | 462 | 0.322 | 0.4217 | Yes |
| 68 | P2RX7 |  |  | 473 | 0.319 | 0.4249 | Yes |
| 69 | SERINC5 |  |  | 477 | 0.318 | 0.4288 | Yes |
| 70 | AASS |  |  | 494 | 0.313 | 0.4312 | Yes |
| 71 | TM7SF3 |  |  | 508 | 0.308 | 0.4339 | Yes |
| 72 | EDNRB |  |  | 519 | 0.306 | 0.4369 | Yes |
| 73 | THY1 |  |  | 546 | 0.299 | 0.4379 | Yes |
| 74 | MAP2 |  |  | 549 | 0.298 | 0.4417 | Yes |
| 75 | SLC22A17 |  |  | 550 | 0.297 | 0.4457 | Yes |
| 76 | ITM2C |  |  | 551 | 0.297 | 0.4497 | Yes |
| 77 | SAR1B |  |  | 562 | 0.295 | 0.4526 | Yes |
| 78 | NTRK2 |  |  | 587 | 0.288 | 0.4537 | Yes |
| 79 | PLOD2 |  |  | 613 | 0.282 | 0.4547 | Yes |
| 80 | IL6ST |  |  | 627 | 0.279 | 0.4569 | Yes |
| 81 | PRKCA |  |  | 652 | 0.275 | 0.4579 | Yes |
| 82 | SLC2A13 |  |  | 664 | 0.272 | 0.4603 | Yes |
| 83 | MPZL1 |  |  | 666 | 0.272 | 0.4639 | Yes |
| 84 | DNER |  |  | 671 | 0.270 | 0.4671 | Yes |
| 85 | LRP1B |  |  | 676 | 0.269 | 0.4702 | Yes |
| 86 | TNK2 |  |  | 681 | 0.268 | 0.4734 | Yes |
| 87 | GOLIM4 |  |  | 699 | 0.262 | 0.4750 | Yes |
| 88 | SDC3 |  |  | 728 | 0.255 | 0.4752 | Yes |
| 89 | CADM2 |  |  | 739 | 0.253 | 0.4775 | Yes |
| 90 | DOCK10 |  |  | 751 | 0.250 | 0.4796 | Yes |
| 91 | GPR155 |  |  | 785 | 0.244 | 0.4791 | Yes |
| 92 | IDH1 |  |  | 793 | 0.242 | 0.4816 | Yes |
| 93 | DHCR24 |  |  | 795 | 0.242 | 0.4847 | Yes |
| 94 | OLIG1 |  |  | 809 | 0.239 | 0.4865 | Yes |
| 95 | NT5E |  |  | 834 | 0.235 | 0.4869 | Yes |
| 96 | SPATA6 |  |  | 852 | 0.232 | 0.4880 | Yes |
| 97 | ZNF37A |  |  | 870 | 0.230 | 0.4892 | Yes |
| 98 | TMEM206 |  |  | 886 | 0.228 | 0.4905 | Yes |
| 99 | IL1RAP |  |  | 890 | 0.227 | 0.4933 | Yes |
| 100 | PTPRE |  |  | 893 | 0.227 | 0.4961 | Yes |
| 101 | PHLDB1 |  |  | 906 | 0.224 | 0.4978 | Yes |
| 102 | NTRK3 |  |  | 908 | 0.224 | 0.5007 | Yes |
| 103 | SSR1 |  |  | 922 | 0.221 | 0.5022 | Yes |
| 104 | LAMA4 |  |  | 931 | 0.220 | 0.5042 | Yes |
| 105 | MRPL32 |  |  | 944 | 0.219 | 0.5058 | Yes |
| 106 | ILDR2 |  |  | 965 | 0.215 | 0.5064 | Yes |
| 107 | PDE4B |  |  | 968 | 0.215 | 0.5091 | Yes |
| 108 | NXPH1 |  |  | 990 | 0.212 | 0.5095 | Yes |
| 109 | PLP1 |  |  | 1022 | 0.208 | 0.5088 | Yes |
| 110 | SNX22 |  |  | 1026 | 0.208 | 0.5112 | Yes |
| 111 | ST3GAL5 |  |  | 1032 | 0.206 | 0.5135 | Yes |
| 112 | C1orf61 |  |  | 1057 | 0.204 | 0.5135 | Yes |
| 113 | LEPROT |  |  | 1072 | 0.202 | 0.5146 | Yes |
| 114 | SLC35F1 |  |  | 1092 | 0.199 | 0.5151 | Yes |
| 115 | LRRN1 |  |  | 1103 | 0.198 | 0.5166 | Yes |
| 116 | NAPEPLD |  |  | 1130 | 0.194 | 0.5162 | Yes |
| 117 | CHRM3 |  |  | 1132 | 0.194 | 0.5187 | Yes |
| 118 | NLGN4X |  |  | 1136 | 0.194 | 0.5210 | Yes |
| 119 | GLCCI1 |  |  | 1172 | 0.188 | 0.5195 | Yes |
| 120 | NTN1 |  |  | 1174 | 0.188 | 0.5219 | Yes |
| 121 | CTTNBP2 |  |  | 1249 | 0.179 | 0.5158 | No |
| 122 | SEMA6D |  |  | 1286 | 0.173 | 0.5140 | No |
| 123 | DNM3 |  |  | 1294 | 0.172 | 0.5155 | No |
| 124 | NLGN1 |  |  | 1305 | 0.170 | 0.5167 | No |
| 125 | GLDC |  |  | 1341 | 0.167 | 0.5149 | No |
| 126 | ANGPTL2 |  |  | 1391 | 0.162 | 0.5115 | No |
| 127 | SULF2 |  |  | 1404 | 0.161 | 0.5123 | No |
| 128 | LCORL |  |  | 1413 | 0.160 | 0.5135 | No |
| 129 | BMP7 |  |  | 1419 | 0.160 | 0.5151 | No |
| 130 | TENM1 |  |  | 1479 | 0.155 | 0.5104 | No |
| 131 | LPP |  |  | 1489 | 0.154 | 0.5114 | No |
| 132 | CLDND1 |  |  | 1501 | 0.152 | 0.5122 | No |
| 133 | TMEFF2 |  |  | 1546 | 0.147 | 0.5091 | No |
| 134 | CASK |  |  | 1617 | 0.140 | 0.5030 | No |
| 135 | CSPG5 |  |  | 1670 | 0.136 | 0.4988 | No |
| 136 | REEP3 |  |  | 1671 | 0.136 | 0.5006 | No |
| 137 | EPN2 |  |  | 1688 | 0.134 | 0.5006 | No |
| 138 | NTM |  |  | 1729 | 0.131 | 0.4978 | No |
| 139 | ZNF462 |  |  | 1814 | 0.126 | 0.4898 | No |
| 140 | CFLAR |  |  | 1828 | 0.125 | 0.4900 | No |
| 141 | KCND3 |  |  | 1838 | 0.123 | 0.4906 | No |
| 142 | UNC80 |  |  | 1861 | 0.122 | 0.4897 | No |
| 143 | RFTN2 |  |  | 1867 | 0.121 | 0.4908 | No |
| 144 | CSGALNACT1 |  |  | 1872 | 0.121 | 0.4920 | No |
| 145 | PCDH17 |  |  | 1889 | 0.120 | 0.4917 | No |
| 146 | NLGN3 |  |  | 1947 | 0.115 | 0.4867 | No |
| 147 | GCC2 |  |  | 1949 | 0.115 | 0.4882 | No |
| 148 | RALGPS2 |  |  | 1955 | 0.115 | 0.4891 | No |
| 149 | WSCD1 |  |  | 1980 | 0.113 | 0.4879 | No |
| 150 | TNRC6C |  |  | 2029 | 0.110 | 0.4839 | No |
| 151 | CALCRL |  |  | 2109 | 0.105 | 0.4762 | No |
| 152 | DSCAM |  |  | 2224 | 0.096 | 0.4643 | No |
| 153 | CHST3 |  |  | 2253 | 0.094 | 0.4624 | No |
| 154 | GRB14 |  |  | 2398 | 0.086 | 0.4469 | No |
| 155 | KANK1 |  |  | 2429 | 0.083 | 0.4446 | No |
| 156 | RNF144A |  |  | 2480 | 0.081 | 0.4399 | No |
| 157 | SHISA9 |  |  | 2493 | 0.080 | 0.4396 | No |
| 158 | UIMC1 |  |  | 2512 | 0.078 | 0.4386 | No |
| 159 | KLHL42 |  |  | 2555 | 0.076 | 0.4348 | No |
| 160 | FIBIN |  |  | 2599 | 0.074 | 0.4308 | No |
| 161 | ITPR2 |  |  | 2803 | 0.063 | 0.4083 | No |
| 162 | PHYHIPL |  |  | 2821 | 0.062 | 0.4071 | No |
| 163 | EPB41L2 |  |  | 2855 | 0.060 | 0.4041 | No |
| 164 | ETV1 |  |  | 2867 | 0.059 | 0.4037 | No |
| 165 | PEAK1 |  |  | 2939 | 0.056 | 0.3962 | No |
| 166 | COL11A1 |  |  | 2950 | 0.055 | 0.3958 | No |
| 167 | RNF157 |  |  | 2955 | 0.055 | 0.3961 | No |
| 168 | ETV5 |  |  | 2957 | 0.055 | 0.3967 | No |
| 169 | PDGFA |  |  | 2967 | 0.054 | 0.3964 | No |
| 170 | LINGO1 |  |  | 2992 | 0.052 | 0.3944 | No |
| 171 | TCF7L1 |  |  | 3012 | 0.051 | 0.3929 | No |
| 172 | ABAT |  |  | 3020 | 0.051 | 0.3927 | No |
| 173 | E2F3 |  |  | 3048 | 0.050 | 0.3903 | No |
| 174 | ZADH2 |  |  | 3093 | 0.047 | 0.3859 | No |
| 175 | FARP2 |  |  | 3144 | 0.045 | 0.3807 | No |
| 176 | SAMD8 |  |  | 3190 | 0.042 | 0.3761 | No |
| 177 | SFT2D1 |  |  | 3198 | 0.041 | 0.3758 | No |
| 178 | MAP4K5 |  |  | 3258 | 0.038 | 0.3695 | No |
| 179 | SKIL |  |  | 3291 | 0.037 | 0.3663 | No |
| 180 | APCDD1 |  |  | 3295 | 0.037 | 0.3665 | No |
| 181 | ZNF516 |  |  | 3341 | 0.034 | 0.3618 | No |
| 182 | TCF7L2 |  |  | 3356 | 0.033 | 0.3606 | No |
| 183 | HIP1 |  |  | 3382 | 0.032 | 0.3582 | No |
| 184 | FOXN2 |  |  | 3403 | 0.032 | 0.3563 | No |
| 185 | LRRK2 |  |  | 3432 | 0.030 | 0.3535 | No |
| 186 | NUP50 |  |  | 3457 | 0.029 | 0.3511 | No |
| 187 | DOCK1 |  |  | 3542 | 0.025 | 0.3417 | No |
| 188 | ARNT2 |  |  | 3544 | 0.025 | 0.3420 | No |
| 189 | CA10 |  |  | 3556 | 0.025 | 0.3410 | No |
| 190 | KLHL5 |  |  | 3580 | 0.024 | 0.3387 | No |
| 191 | SIRT2 |  |  | 3589 | 0.024 | 0.3381 | No |
| 192 | UBE3B |  |  | 3615 | 0.023 | 0.3355 | No |
| 193 | BPNT1 |  |  | 3665 | 0.021 | 0.3302 | No |
| 194 | MED11 |  |  | 3693 | 0.020 | 0.3273 | No |
| 195 | PCMTD2 |  |  | 3707 | 0.020 | 0.3261 | No |
| 196 | LRRN3 |  |  | 3720 | 0.019 | 0.3250 | No |
| 197 | BLM |  |  | 3771 | 0.017 | 0.3194 | No |
| 198 | SPRY4 |  |  | 3821 | 0.015 | 0.3140 | No |
| 199 | MAMLD1 |  |  | 3895 | 0.012 | 0.3057 | No |
| 200 | ARHGAP42 |  |  | 4172 | -0.000 | 0.2739 | No |
| 201 | CNTN1 |  |  | 4200 | -0.001 | 0.2708 | No |
| 202 | MAP3K1 |  |  | 4259 | -0.004 | 0.2642 | No |
| 203 | SOX2 |  |  | 4271 | -0.005 | 0.2630 | No |
| 204 | RAP2C |  |  | 4293 | -0.006 | 0.2606 | No |
| 205 | TNS3 |  |  | 4375 | -0.009 | 0.2514 | No |
| 206 | ZC3HAV1 |  |  | 4394 | -0.010 | 0.2495 | No |
| 207 | PKP4 |  |  | 4407 | -0.010 | 0.2482 | No |
| 208 | KIAA0232 |  |  | 4475 | -0.013 | 0.2407 | No |
| 209 | SATB1 |  |  | 4505 | -0.014 | 0.2375 | No |
| 210 | C2orf80 |  |  | 4540 | -0.016 | 0.2338 | No |
| 211 | PIK3R1 |  |  | 4545 | -0.016 | 0.2336 | No |
| 212 | SEPT8 |  |  | 4589 | -0.018 | 0.2288 | No |
| 213 | CPEB2 |  |  | 4705 | -0.022 | 0.2159 | No |
| 214 | IQSEC1 |  |  | 4764 | -0.024 | 0.2095 | No |
| 215 | SHC3 |  |  | 4768 | -0.025 | 0.2095 | No |
| 216 | ACTN4 |  |  | 4829 | -0.027 | 0.2029 | No |
| 217 | SPRY1 |  |  | 4844 | -0.027 | 0.2017 | No |
| 218 | NOC3L |  |  | 4847 | -0.027 | 0.2018 | No |
| 219 | ANKS1B |  |  | 5003 | -0.033 | 0.1844 | No |
| 220 | MYO10 |  |  | 5109 | -0.037 | 0.1728 | No |
| 221 | FBXO7 |  |  | 5143 | -0.038 | 0.1695 | No |
| 222 | STXBP3 |  |  | 5173 | -0.039 | 0.1667 | No |
| 223 | APBB2 |  |  | 5184 | -0.039 | 0.1661 | No |
| 224 | TAOK3 |  |  | 5204 | -0.041 | 0.1644 | No |
| 225 | PGM3 |  |  | 5211 | -0.041 | 0.1643 | No |
| 226 | KIF13A |  |  | 5331 | -0.046 | 0.1512 | No |
| 227 | CAT |  |  | 5358 | -0.047 | 0.1488 | No |
| 228 | ABI1 |  |  | 5375 | -0.048 | 0.1476 | No |
| 229 | KAT2B |  |  | 5388 | -0.048 | 0.1469 | No |
| 230 | STRN |  |  | 5405 | -0.049 | 0.1457 | No |
| 231 | TP53INP1 |  |  | 5454 | -0.050 | 0.1409 | No |
| 232 | TAF2 |  |  | 5457 | -0.051 | 0.1413 | No |
| 233 | PHLPP1 |  |  | 5506 | -0.053 | 0.1365 | No |
| 234 | COPB2 |  |  | 5597 | -0.057 | 0.1269 | No |
| 235 | B9D1 |  |  | 5602 | -0.058 | 0.1272 | No |
| 236 | CHIC2 |  |  | 5655 | -0.060 | 0.1220 | No |
| 237 | ALDH6A1 |  |  | 5682 | -0.061 | 0.1199 | No |
| 238 | ATCAY |  |  | 5683 | -0.061 | 0.1207 | No |
| 239 | QKI |  |  | 5715 | -0.062 | 0.1180 | No |
| 240 | KHSRP |  |  | 5808 | -0.066 | 0.1082 | No |
| 241 | SASH1 |  |  | 5850 | -0.067 | 0.1044 | No |
| 242 | GATM |  |  | 6035 | -0.076 | 0.0842 | No |
| 243 | WASF1 |  |  | 6054 | -0.077 | 0.0832 | No |
| 244 | CUTC |  |  | 6093 | -0.079 | 0.0799 | No |
| 245 | PDLIM5 |  |  | 6127 | -0.081 | 0.0772 | No |
| 246 | ATP6V0E1 |  |  | 6138 | -0.082 | 0.0771 | No |
| 247 | C1orf21 |  |  | 6205 | -0.085 | 0.0707 | No |
| 248 | FRMD6 |  |  | 6281 | -0.089 | 0.0632 | No |
| 249 | FGF12 |  |  | 6306 | -0.091 | 0.0617 | No |
| 250 | HIPK2 |  |  | 6320 | -0.091 | 0.0614 | No |
| 251 | LIMA1 |  |  | 6339 | -0.092 | 0.0606 | No |
| 252 | DENND5A |  |  | 6342 | -0.092 | 0.0616 | No |
| 253 | SMAD5 |  |  | 6355 | -0.093 | 0.0615 | No |
| 254 | TJP1 |  |  | 6500 | -0.101 | 0.0463 | No |
| 255 | OLIG2 |  |  | 6595 | -0.108 | 0.0369 | No |
| 256 | UGDH |  |  | 6647 | -0.111 | 0.0325 | No |
| 257 | GUCD1 |  |  | 6650 | -0.111 | 0.0338 | No |
| 258 | FNTA |  |  | 6659 | -0.111 | 0.0343 | No |
| 259 | FYN |  |  | 6823 | -0.123 | 0.0172 | No |
| 260 | BAALC |  |  | 6848 | -0.125 | 0.0161 | No |
| 261 | NDUFAF2 |  |  | 6896 | -0.129 | 0.0124 | No |
| 262 | ARL4A |  |  | 6930 | -0.131 | 0.0104 | No |
| 263 | ZEB1 |  |  | 6944 | -0.132 | 0.0107 | No |
| 264 | ZMAT3 |  |  | 6960 | -0.134 | 0.0108 | No |
| 265 | CDCA7L |  |  | 7034 | -0.140 | 0.0043 | No |
| 266 | CAMSAP2 |  |  | 7038 | -0.140 | 0.0058 | No |
| 267 | EVI5 |  |  | 7077 | -0.143 | 0.0034 | No |
| 268 | ZCCHC24 |  |  | 7078 | -0.143 | 0.0053 | No |
| 269 | RAP2A |  |  | 7134 | -0.148 | 0.0010 | No |
| 270 | GNB4 |  |  | 7251 | -0.162 | -0.0102 | No |
| 271 | DDAH1 |  |  | 7257 | -0.162 | -0.0086 | No |
| 272 | DUSP6 |  |  | 7258 | -0.162 | -0.0064 | No |
| 273 | BCAT1 |  |  | 7285 | -0.166 | -0.0072 | No |
| 274 | SEPT2 |  |  | 7297 | -0.167 | -0.0062 | No |
| 275 | CISD1 |  |  | 7408 | -0.182 | -0.0164 | No |
| 276 | SPRY2 |  |  | 7461 | -0.187 | -0.0198 | No |
| 277 | PPP2R2B |  |  | 7511 | -0.192 | -0.0229 | No |
| 278 | RSU1 |  |  | 7513 | -0.192 | -0.0204 | No |
| 279 | UBL3 |  |  | 7525 | -0.194 | -0.0191 | No |
| 280 | STARD7 |  |  | 7579 | -0.201 | -0.0225 | No |
| 281 | GLTP |  |  | 7609 | -0.205 | -0.0230 | No |
| 282 | TNFRSF21 |  |  | 7627 | -0.208 | -0.0222 | No |
| 283 | PCBP4 |  |  | 7643 | -0.210 | -0.0211 | No |
| 284 | WDR83OS |  |  | 7685 | -0.215 | -0.0229 | No |
| 285 | SH3D19 |  |  | 7694 | -0.217 | -0.0209 | No |
| 286 | RAB9A |  |  | 7740 | -0.224 | -0.0230 | No |
| 287 | TRIB2 |  |  | 7741 | -0.224 | -0.0200 | No |
| 288 | OCIAD2 |  |  | 7875 | -0.249 | -0.0320 | No |
| 289 | PDE1C |  |  | 7890 | -0.253 | -0.0302 | No |
| 290 | DENR |  |  | 7954 | -0.266 | -0.0338 | No |
| 291 | ACP1 |  |  | 7982 | -0.272 | -0.0333 | No |
| 292 | PRDX3 |  |  | 8005 | -0.277 | -0.0320 | No |
| 293 | SEPT7 |  |  | 8018 | -0.279 | -0.0297 | No |
| 294 | PMP2 |  |  | 8058 | -0.288 | -0.0303 | No |
| 295 | RPS27L |  |  | 8126 | -0.302 | -0.0339 | No |
| 296 | SNX7 |  |  | 8188 | -0.321 | -0.0366 | No |
| 297 | MT3 |  |  | 8279 | -0.348 | -0.0422 | No |
| 298 | CNP |  |  | 8294 | -0.352 | -0.0391 | No |
| 299 | LITAF |  |  | 8324 | -0.361 | -0.0376 | No |
| 300 | GNG12 |  |  | 8328 | -0.362 | -0.0330 | No |
| 301 | CCND1 |  |  | 8346 | -0.368 | -0.0300 | No |
| 302 | LDHB |  |  | 8372 | -0.379 | -0.0277 | No |
| 303 | NDUFB3 |  |  | 8447 | -0.406 | -0.0308 | No |
| 304 | LYPD1 |  |  | 8476 | -0.422 | -0.0283 | No |
| 305 | KHDRBS3 |  |  | 8542 | -0.452 | -0.0297 | No |
| 306 | NME1 |  |  | 8573 | -0.471 | -0.0267 | No |
| 307 | MARCKS |  |  | 8667 | -0.537 | -0.0302 | No |
| 308 | SLC25A5 |  |  | 8681 | -0.546 | -0.0243 | No |
| 309 | PHLDA1 |  |  | 8805 | -0.655 | -0.0296 | No |
| 310 | FABP5 |  |  | 8829 | -0.684 | -0.0230 | No |
| 311 | RAB31 |  |  | 8831 | -0.687 | -0.0138 | No |
| 312 | DSTN |  |  | 8885 | -0.752 | -0.0098 | No |
| 313 | DBI |  |  | 8895 | -0.764 | -0.0005 | No |
| 314 | S100B |  |  | 8908 | -0.784 | 0.0088 | No |
Table: GSEA details [plain text format]

  

Fig 2: RICHARDS\_NOWAKOWSKI\_SCIENCE\_2017\_OPC\_UPREG: Random ES distribution      
 Gene set null distribution of ES for **RICHARDS\_NOWAKOWSKI\_SCIENCE\_2017\_OPC\_UPREG**
